# *De Novo* Assembly of a Bell Pepper Endornavirus Genome Sequence Using RNA Sequencing Data

**DOI:** 10.1128/genomeA.00061-15

**Published:** 2015-03-19

**Authors:** Yeonhwa Jo, Hoseng Choi, Won Kyong Cho

**Affiliations:** Department of Agricultural Biotechnology, College of Agriculture and Life Sciences, Seoul National University, Seoul, Republic of Korea

## Abstract

The genus *Endornavirus* is a double-stranded RNA virus that infects a wide range of hosts. In this study, we report on the *de novo* assembly of a bell pepper endornavirus genome sequence by RNA sequencing (RNA-Seq). Our result demonstrates the successful application of RNA-Seq to obtain a complete viral genome sequence from the transcriptome data.

## GENOME ANNOUNCEMENT

The members of the genus *Endornavirus*, of the family *Endornaviridae*, have been identified in a wide range of hosts, such as plants, fungi, and oomycetes (1, 2). In general, the genomes of endornaviruses are composed of a double-stranded RNA of 9.8 to 17.6 kb in length that encodes a single polyprotein (3). In particular, they do not form true virions, and they present in the host at low copy numbers (2). In addition, they are vertically transmitted by seeds and do not cause any observable disease symptoms (2). So far, the genomes for several different endornaviruses have been sequenced (3).

In our search for endornaviruses infecting various plant species, we found a long novel transcript (accession no. JW157405.1) from the transcriptome shotgun assembly (TSA) sequence database that showed a strong sequence identity to bell pepper endornavirus (4). The transcriptome was derived from a previous study in which transcriptome analysis was performed for three different pepper cultivars by next-generation sequencing (5). The mRNA libraries were sequenced by Illumina GAIIx and *de novo* assembled by combining three different assembly methods, Velvet, CLC Genomics Workbench, and CAP3 (6). To confirm *de novo* transcriptome assembly, we downloaded the raw data for the pepper cultivar Maor from the NCBI Short Read Archive (SRA) database and performed *de novo* transcriptome assembly using the Trinity method (7). A novel transcript similar to bell pepper endornavirus was identified. This result suggests the successful *de novo* assembly of a viral genome regardless of the transcriptome assembly method. After alignment of the obtained transcript on the bell pepper endornavirus reference sequence (GenBank accession no. NC_015781.2), we found that an adenine was inserted in the middle of the assembled transcript (at nucleotide [nt] 7633), which led to a frameshift. After removing the adenine nucleotide, the complete open reading frame was obtained. We named the newly identified endornavirus bell pepper endornavirus isolate Maor. Its complete genome is 14,659 bp long, which is 69 bp shorter than the reference genome. In particular, the 5′- and 3′-terminal noncoding regions of the obtained viral genome are 61 bp and 8 bp shorter, respectively, than those of the reference genome. In addition, the BLAST result showed that the genome of bell pepper endornavirus isolate Maor is 88% identical to that of the reference genome. Specifically, the open reading frame region is highly variable between the two viral genomes. The G+C content for isolate Maor is 41.01%, while that of the reference genome is 40.41%. The bell pepper endornavirus isolate Maor encodes a polyprotein 4,815 amino acids in length and containing four viral domains, including methyltransferase, helicase, glycosyltransferase, and RNA-dependent RNA polymerase. Taken together, we demonstrated the successful application of *de novo* assembly by RNA sequencing (RNA-Seq) to reveal a complete viral genome sequence from the transcriptome data.

### Nucleotide sequence accession number.

The genome sequence of bell pepper endornavirus isolate Maor has been deposited in GenBank under the accession no. KP455654.
